# Valaciclovir therapy for secondary suppression of immune response to herpesviruses: An exploratory study

**DOI:** 10.1371/journal.ppat.1013803

**Published:** 2025-12-29

**Authors:** Kirsty McGee, Alex Dowell, Sarah Lauder, Jusnara Begum, Odette Chagoury, Wayne Croft, Lee Middleton, Yongzhong Sun, Jianmin Zuo, Richard McManus, Paul Moss

**Affiliations:** 1 School of Infection, Inflammation and Immunology, College of Medicine and Health, University of Birmingham, Birmingham, United Kingdom; 2 National Institute for Health and Care Research (NIHR) Birmingham Biomedical Research Centre, Birmingham, United Kingdom; 3 Department of Immunology, Cardiff University, Cardiff, United Kingdom; 4 Qatar Metabolic Institute, Hamad Medical Corporation, Doha, Qatar; 5 Weill Cornell Medicine-Qatar, Doha, Qatar; 6 School of Health Sciences, University of Birmingham, Birmingham, United Kingdom; 7 Brighton and Sussex Medical School, University of Brighton and University of Sussex, Brighton, United Kingdom; 8 University Hospital Birmingham NHS Foundation Trust, Birmingham, United Kingdom; Thomas Jefferson University, UNITED STATES OF AMERICA

## Abstract

Herpesviruses establish a state of persistent infection which is suppressed by sustained virus-specific immune control. The magnitude of this immune response can increase with age and lead to attrition of immune reserve against other pathogens. Approaches which suppress herpesvirus-specific immunity may therefore have the potential to improve general immune function. Anti-retroviral therapy for HIV leads to a reduction in HIV viral antigen and has been shown to mediate a secondary attenuation of the HIV-specific immune response. As such, we assessed if treatment with valaciclovir could suppress the immune response against cytomegalovirus and Epstein Barr Virus in donors aged >65 years. Medication was given at 3 different doses up to a maximum of 4gm/day for 6 months and humoral and cellular profiles were assessed over 12 months. Anti-viral therapy did not impact on the magnitude or phenotype of the humoral or cellular virus-specific immune response during the study period. Treatment also had no impact of physical or mental quality of life assessment. These data show that valaciclovir treatment, at this dose and treatment duration, does not attenuate the CMV or EBV-specific immune response in this age group.

## Author summary

Cytomegalovirus (CMV) is a herpesvirus that infects most people during their lifetime. The virus can never be eradicated after infection and its replication must be suppressed by sustained immune pressure throughout life. This virus-specific immune response becomes very large in older people and may limit the ability of the immune system to make effective immune responses against other infections. Here we performed a clinical trial in which we treated healthy older people for six months with the antiviral drug valaciclovir to see if suppression of CMV would subsequently attenuate the CMV-specific immune response and improve well being. This approach did not lead to a reduction in CMV-specific immune response and had no impact on health measurements. As such, alternative approaches such as extended duration therapy or alternative anti-viral drugs are required in future studies to assess potential suppression of herpesvirus-specific immunity.

## Introduction

Human herpesviruses (HHV) are a family of eight dsDNA viruses that establish persistent infection and are widely prevalent. HHV have evolved a range of mechanisms to mediate latent and lytic replication and the host immune response is critical for control of viral replication. Cytomegalovirus (CMV; HHV-5) is particularly immunodominant and the cellular immune response against CMV can represent over 10% of the peripheral T cell pool [1,2]. This increment in the T cell memory pool leads to relative suppression of the naïve repertoire that is required for immunity against novel pathogens [3–5]. The magnitude of the adaptive immune response against CMV increases with age [6] and positive CMV serostatus has been associated with the development of immune senescence and impaired immune response to influenza vaccination [7,8]. There is therefore interest in approaches that may act to suppress the magnitude of the CMV-specific immune response and thereby support rejuvenation of immune function. A particular feature of the association between persistent CMV infection and poor health is that the magnitude of the CMV-specific immune response is itself a determinant of risk [9–13]. The reason for this is not clear but may represent an inflammatory burden arising from inflated virus-specific immunity driven by subclinical virus replication [14,15]. An approach that could act to reduce the magnitude of virus-specific immune responses could thus hold clinical value.

Epstein Barr Virus infection (EBV; HHV-4) is also highly prevalent in the adult population and associated with multiple sclerosis and several malignant disorders [16]. The immune response against EBV is directed against a range of lytic and latent proteins and the magnitude and phenotype of this response varies according to epitope specificity [17]. The inflammatory and clinical burden arising from sustained EBV-specific immunity is currently uncertain but there is increasing interest in the potential importance of EBV carriage on heterologous immunity [18–20].

Virus-specific immune responses are boosted by intermittent stimulation by viral antigen [21]. Indeed, the magnitude of CMV-specific immunity is related directly to latent viral load [22]. Given this observation, suppression of viral load by anti-viral medication may attenuate the magnitude of adaptive immunity. This has been observed clearly in the setting of HIV infection where HIV-specific cellular immune responses are markedly reduced following the introduction of anti-retroviral therapy [23]. In this setting, the HIV specific CD8^+^ T cell count is seen to decline with an initial half-life of around 39 weeks [24], a value comparable with t_1/2_ of memory T cells [25].^.^Herpesvirus-specific T cells have comparable rates of turnover [26–28] and anti-viral therapy might therefore also hold potential for modulation of the immune response against viruses such as CMV or EBV. A range of anti-viral medications have been developed against CMV and EBV although the side effect profile of agents such as ganciclovir, foscarnet and cidofovir is such that the risk-benefit ratio for their use in immune modulation prophylaxis is uncertain. Letermovir combines efficacy with good safety profile but its use has been limited largely to patients undergoing transplantation [29]. Aciclovir is an effective and very well tolerated anti-viral medication with activity against a range of herpesviruses. Valaciclovir is the l-valyl ester of aciclovir and is licensed in some countries for suppression of CMV reactivation following renal transplantation at a dose of 8gm/day, although a regime of 3gm daily has also shown utility [30]. Valaciclovir can also reduce the frequency of EBV-infected B cells [31]. Chronic valaciclovir administration has been shown to markedly suppress CMV-specific T cells in murine models and enhance reconstitution of the naïve T cell pool [32]. However, to our knowledge, valaciclovir has not yet been assessed for its ability to suppress CMV viral load or modulate HHV-specific immunity in immunocompetent donors.

Here we undertook a clinical study to determine the potential impact of valaciclovir treatment on CMV and EBV viral load and virus-specific immune response during and following 6 months of therapy. The findings indicate a limited impact of this treatment regimen on modulation of the CMV and EBV-specific immune response.

## Results

### Valaciclovir therapy does not reduce CMV or EBV viral load

41 donors were recruited to the study with a median age of 72 years. Donors were allocated randomly into four groups comprising either no treatment (n = 10) or valaciclovir at a dose of 500mg twice daily (n = 7), 1gm twice daily (n = 10) or 1gm four times daily (n = 11) for 6 months (S1 Table). 3 donors assigned to the 500mg dose declined study entry after randomisation leaving 38 for whom results are presented. 58% of donors were male and demographic factors were evenly distributed across groups (Table 1).

**Table 1:** Demographic features of treatment groups.

		No treatment (n = 10)	500mg valaciclovir b.d. (n = 7)	1000mg valaciclovir b.d. (n = 10)	1000mg valaciclovir q.d.s (n = 11)	Total (n = 38)
**Minimisation variables**						
**Age at time of randomization (years), n (%)**	65-74	7 (70)	4 (57)	6 (60)	7 (64)	24 (63)
	≥ 75	3 (30)	3 (43)	4 (40)	4 (36)	14 (37)
	Mean (SD)	73.3 (6.0)	73.3 (5.3)	73.4 (4.7)	73.6 (6.9)	73.4 (5.6)
	Median [IQR]	72.2 [68.5-77.5]	73.4 [69.7-77.0]	72.1 [69.0-78.2]	72.1 [67.0-80.0]	72.4 [69.0-77.5]
**Demographic and other baseline variables**						
**Gender, n (%)**	Female	4 (40)	3 (43)	3 (30)	6 (55)	16 (42)
	Male	6 (60)	4 (57)	7 (70)	5 (45)	22 (58)
**Ethnicity, n (%)**	White British	10 (100)	6 (86)	10 (100)	11 (100)	37 (97)
	Indian	0 (0)	1 (14)	0 (0)	0 (0)	1 (3)
**BMI (kg/m**^**2**^)	Mean (SD)	28.2 (3.8)	27.5 (4.0)	27.1 (3.0)	25.8 (4.3)	27.1 (3.8)
	Median [IQR]	29.2 [26.6-29.7]	29.0 [23.5-31.6]	27.2 [24.4-29.8]	25.5 [23.0-27.8]	26.9 [23.5-29.8]
	Missing	1	0	0	0	1
**Medical history, n (%)**	Yes	5 (50)	2 (29)	5 (50)	3 (27)	15 (39)
	No	5 (50)	5 (71)	5 (50)	8 (73)	23 (61)
**Current medication, n (%)**	Yes	3 (30)	2 (29)	3 (30)	4 (36)	12 (32)
	No	7 (70)	5 (71)	7 (70)	7 (64)	26 (68)
**Flu vaccination, n (%)**	Yes	5 (50)	3 (43)	5 (50)	2 (18)	15 (39)
	No	5 (50)	4 (57)	5 (50)	9 (82)	23 (61)
**Pneumonia vaccination, n (%)**	Yes	4 (40)	0 (0)	2 (20)	1 (9)	7 (18)
	No	6 (60)	7 (100)	8 (80)	10 (91)	31 (82)
**Shingles vaccination, n (%)**	Yes	0 (0)	0 (0)	0 (0)	0 (0)	0 (0)
	No	10 (100)	7 (100)	10 (100)	11 (100)	38 (100)
**EQ-5D-5L score**	Mean (SD)	0.92 (0.07)	0.96 (0.05)	0.90 (0.09)	0.89 (0.14)	0.91 (0.10)
	Median [IQR]	0.92 [0.89-1.0]	1.0 [0.89-1.0]	0.89 [0.84-0.94]	0.94 [0.82-1.0]	0.94 [0.88-1.0]
	Missing	0	0	1	0	1
**SF36 (physical component score)**	Mean (SD)	44.6 (13.5)	46.7 (3.4)	47.7 (5.5)	42.9 (15.8)	45.1 (11.5)
	Median [IQR]	49.9 [43.2-54.5]	46.9 [43.4-49.1]	49.3 [45.8-51.7]	51.3 [33.6-53.2]	49.5 [43.4-51.7]
	Missing	0	0	4	1	5
**SF36 (mental component score)**	Mean (SD)	60.9 (4.2)	59.7 (5.0)	61.7 (3.1)	58.8 (6.7)	60.2 (5.0)
	Median [IQR]	61.4 [57.2-63.7]	61.9 [54.6-63.1]	61.8 [60.4-64.2]	59.4 [57.6-62.0]	61.1 [57.6-63.1]
	Missing	0	0	4	1	5

**Fig 1:**
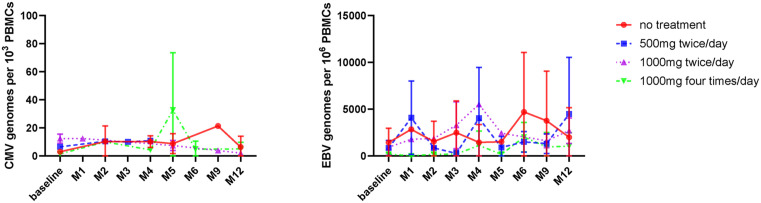
Valaciclovir therapy does not suppress CMV or EBV viral load. EBV and CMV virus load in PBMC was measured using Q-PCR. Copy number of β2m was used to determine the cell input in each sample. Data shown as copy numbers per thousands PBMCs (CMV) and per million PBMCs (EBV); analysis in all donors in study.

Initial work focussed on the potential impact of valaciclovir treatment on the viral load of CMV or EBV within peripheral blood. Baseline viral load measurement showed variation in median values between subgroups at study entry. CMV viral load was broadly stable over time whilst more variation was observed in EBV values. However, anti-viral therapy had no impact on viral load either during the treatment period or within follow up (Fig 1).

### Valaciclovir therapy does not alter CMV-specific antibody titre or the number of CMV-specific T cells

We next went on to determine the impact of valaciclovir therapy on the adaptive humoral and cellular immune response to CMV.

**Fig 2:**
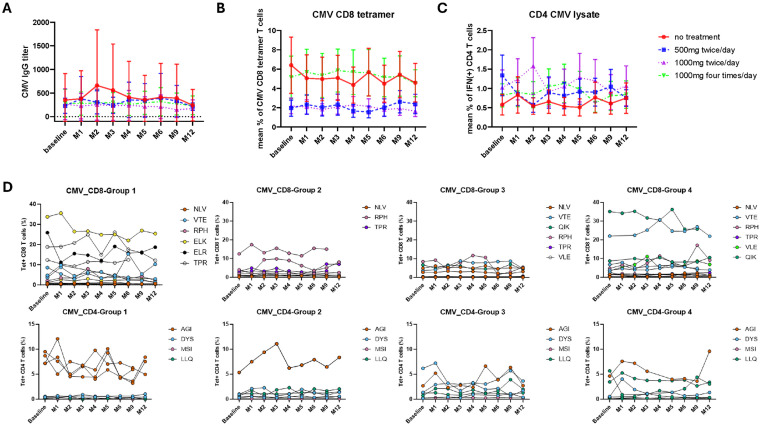
CMV-specific antibody and T cell immune response is not reduced by valaciclovir therapy. A. CMV antibody titre measured by ELISA (n = 38). B. CMV-specific CD4^+^ T cell response measured by cytokine production following stimulation with viral lysate (n = 38). C. Frequency of CMV-specific CD8^+^ T cells measured by HLA class I peptide tetramers (n = 25). D. Frequency of CD4^+^ and CD8^+^ T cells binding individual HLA-peptide tetramers within each treatment subgroup. Differences in tetramer usage between groups represent assignment according to HLA genotype distribution. Group 1 is the no treatment group, group 2 is treatment group with dose of 500mg twice daily, group 3 with the dose of 1gm twice daily and group 4 with 1gm four times daily.

Baseline CMV-specific antibody titre varied between donors but was stable thereafter within all groups and not impacted by anti-viral therapy (Fig 2A and S1 Table).

CMV-specific CD4^+^ T cells were identified by IFN-γ release following stimulation with CMV viral lysate. Mean values at baseline varied between 0.5% and 1.6% of the CD4^+^ T cell pool [33] and no consistent changes were observed in these values over the study period in the 4 treatment subgroups (Fig 2B). Lysate stimulation was not used to quantify virus-specific CD8 + T cells due to the low efficiency of cross presentation of protein through the HLA class I presentation pathway.

CMV-specific CD8^+^ and CD4^+^ T cells were identified through the use of HLA class I and class II-peptide tetramers. Summation of individual tetramer-specific responses within each donor was used as a measure of the aggregate CMV-specific CD8^+^ T cell response. These values varied at baseline in the different treatment subgroups but overall numbers remained stable over the 12-month study period (Fig 2C). The percentage of T cells bound by individual CMV-specific tetramers was also examined and whilst these values were remarkably stable over the study there was no impact of anti-viral therapy on these values (Fig 2D).

These findings show that anti-viral therapy did not suppress the magnitude of the humoral or cellular immune response against CMV.

### Valaciclovir therapy does not alter EBV-specific antibody titre or EBV-specific T cell number

The impact of valaciclovir therapy was next assessed on the magnitude of the adaptive immune response against EBV.

**Fig 3:**
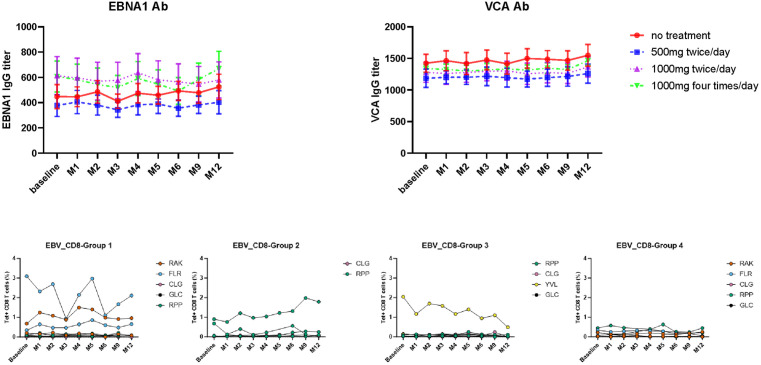
EBV-specific antibody and T cell response is not reduced by valaciclovir therapy. A. Antibody titre against EBNA1 measured by ELISA. Antibody titre against VCA measured by ELISA. **B.** Frequency of EBV-specific CD8^+^ T cells measured by HLA class I peptide tetramers. **C.** Group 1 no treatment; group 2 500mg twice daily; group 3 1gm twice daily; group 4 1gm four times daily.

Antibody titres against EBNA-1 and VCA were measured in donors at each timepoint. Values were very stable over the study period within both the control group and treatment cohorts (Fig 3A).

The CD8^+^ T cell response against EBV was next assessed using HLA class I-peptide tetramers containing immunodominant peptides. The median value of both the aggregate and individual tetramer response varied between groups at study entry and in relation to epitope. However, no significant variation was observed in prospective values over the study period (Fig 3B).

These data show that anti-viral treatment had no impact on the humoral and CD8^+^ T cell response against EBV.

### Valaciclovir therapy does not alter the number of circulating CD28- T cells

CD28 is an important co-stimulatory molecule on T cells and loss of CD28 expression is observed on subpopulations of T cells during clonal expansion and differentiation. CMV or EBV-specific CD8^+^ T cells frequently express a CD28^-^ phenotype whilst CD4^+^CD28^-^ cells are observed almost exclusively within CMV seropositive donors [34]. Given this association, we next enumerated the number of CD28^-^ T cells during the study period.

**Fig 4:**
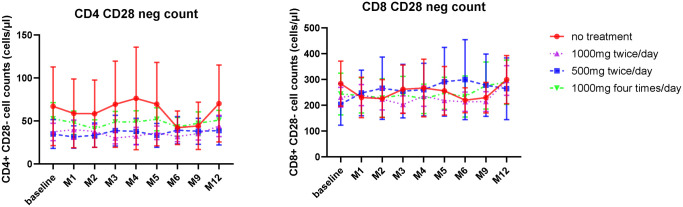
CD28- T cell count is not influenced by valaciclovir therapy. A. CD4^+^CD28^-^ T cell count within study groups (Median +SE). B. CD8^+^CD28^-^ T cell count within study groups (Median +SE).

Variation in the median percentage of CD4^+^CD28^-^ T cells was seen at baseline and reflects the heterogeneity of this value across the population [35]. CD4^+^CD28^-^ T cell number thereafter remained broadly stable during the study period (Fig 4A). A similar profile was observed for the CD8^+^CD28^-^ subset where cell number was not altered during the study treatment period (Fig 4B).

### Anti-viral therapy does not modulate the memory status of phenotype of CMV-specific T cells

**Fig 5:**
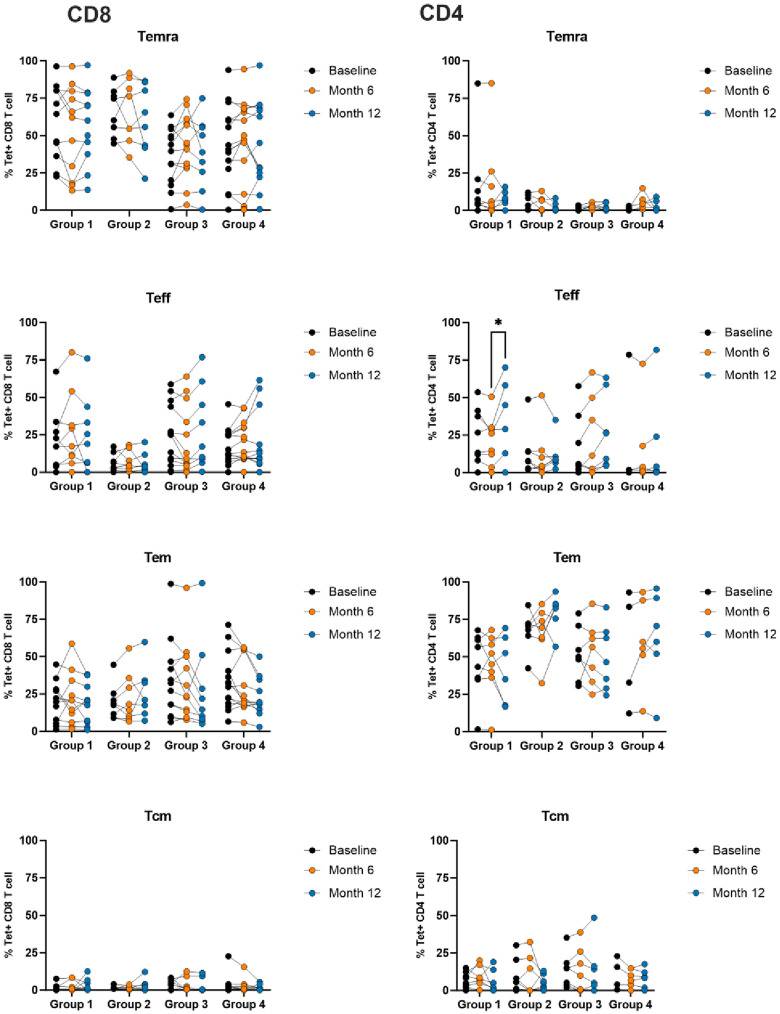
CMV-specific T cell memory profile remains stable despite anti-viral therapy. CMV-specific T cells were identified by HLA-peptide tetramer staining and allocated to TEMRA (Temra), effector (Teff), effector memory (Tem) or central memory (Tcm) subgroups based on CD45, CCR7 and CD28 expression. No significant alteration in distribution was observed during the study period. Group 1 is the no treatment group, group 2 is treatment group with dose of 500mg twice daily, group 3 with the dose of 1gm twice daily and group 4 with 1gm four times daily.

Although the number of CMV and EBV-specific T cells was not reduced by anti-viral therapy we next assessed the effector and memory status of virus-specific T cells to assess if this might be influenced by a potential reduction in viral antigen. CMV-specific CD4^+^ and CD8^+^ T cells were identified by HLA peptide tetramer staining and antibody staining used to differentiate naïve (CD45RO^-^CCR7^+^), central memory (CD45RO^+^CCR7^+^), early effector (CD45RO^+^CCR7^-^CD28^+^), late effector (CD45RO^-^CCR7^-^CD28^-^) and CD45RA^+^ memory (Temra; CD45RA^+^CCR7^-^CD28^-^) populations. CD4^+^ populations were seen to comprise mainly effector or effector memory phenotype whilst CD8^+^ cells had a dominant CD45RA^+^ memory profile (Fig 5). Overall, the relative distribution of virus-specific T cells within these memory subsets was not influenced by anti-viral therapy. The number of total CD4+ and CD8 + memory cells within blood was also determined but did not vary during treatment (S1 Fig).

### Valaciclovir treatment was well tolerated and assessment of physical and mental quality of life remained stable during the study period

**Fig 6:**
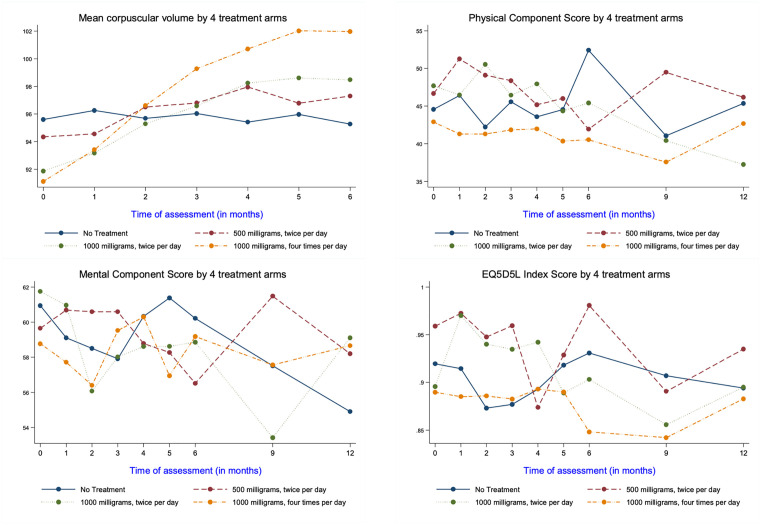
Valaciclovir therapy was not associated with any change in physical or mental score. Physical component score, mental component score, and EQ-5D-5L index were measured at each timepoint. These values remained stable over time within the study groups.

No clinical complications were encountered during the study and medication was well tolerated, in line with the substantial clinical experience with valaciclovir. Baseline laboratory haematological and biochemical values remained stable and the only notable feature was an increase in the mean red cell volume (MCV) whilst on valaciclovir, an association that has been previously reported [36]. This value increased by 3, 6 and 10 fl respectively with the increasing doses of valaciclovir (Fig 6).

Physical and mental component scores, as well as EQ5DS5, were measured at monthly intervals but did not change during the study period (Fig 6).

## Discussion

Immune surveillance of persistent herpesvirus infections requires substantial metabolic investment and can mediate an attritional impact on heterologous immune function. Here we assessed if a 6-month course of valaciclovir therapy, acting to reduce viral load, had the potential to attenuate the cytomegalovirus and Epstein-Barr virus-specific immune response. This treatment was seen to have little impact on viral-specific immune responses and the findings raise several questions in relation to future approaches to attenuate the burden of persistent herpesvirus infections.

Initial studies assessed CMV and EBV viral load which is maintained predominantly within the myeloid and B cell compartments respectively. A notable feature was the substantial baseline heterogeneity of viral load across the study group. Such variation in the setpoint of viral load is well established [37] and is likely to be an important determinant of the magnitude of associated immune response and potential secondary immunopathology. Notwithstanding this baseline heterogeneity, there was no evidence that valaciclovir treatment had an impact on viral load across all study groups.

Valaciclovir inhibits lytic viral replication but does not impact directly on latent carriage, although this may become suppressed over the longer term due to reduction of infectious virus required for reestablishment of latent load [38]. Valaciclovir has proven efficacy against CMV and EBV lytic replication and has been used widely for prevention of CMV reactivation in immune suppressed patients. Given this background it is noteworthy to reflect on why no suppression of viral load was apparent in this study. One factor here may have been that a dose of 8gm/day is recommended to prevent CMV reactivation whilst a maximal daily dose of 4gm/day was used in the study to minimize potential toxicity in healthy volunteers. However, a dose of 3gm/day has been shown to reduce CMV reactivation in immune suppressed patients and should be highly effective in suppressing lytic replication of EBV [38]. The duration of therapy may be a key determinant in subsequent suppression of latent load. Around 1/10,000–1/100,000 B cells are latently infected with EBV in healthy donors and valaciclovir can reduce this burden, albeit with a t_1/2_ of 11 months. Indeed, it has been estimated that a 99% reduction in viral load would potentially be achievable following 6 years of therapy [31]. It is possible that the 6-month duration of therapy within our study may have been too short to have any impact on viral load dynamics. Nevertheless, valaciclovir is extremely well tolerated and can be taken for many years and it may be important to assess the impact of long-term therapy in future studies [39].

Treatment efficacy may also relate to the relative immune competence of the host. A 6-month course of valaciclovir therapy has been shown to mediate a 28% reduction the proportion of CD4^+^CD28^-^ T cells in patients undergoing immunosuppressive therapy for vasculitis and was associated with enhanced immune response to pneumococcal vaccination [40]. Subclinical CMV reactivation is common in this patient group and associates with expansion of the CMV-specific T cell pool. Anti-viral therapy may therefore have more notable impact in this setting.

Assessment of the magnitude of the virus-specific T cell response and antibody titre showed that these were not impacted by anti-viral therapy. No alteration in the phenotype of virus-specific cells was observed although future studies could extend this to include more detailed assessment of cytokine production beyond IFN-γ. Herpes virus-specific T cell responses are boosted strongly by episodes of viral replication [21] and removal of antigenic protein through use of antivirals might have been anticipated to limit immune cell expansion. The dynamics of any potential effect are again of interest. Murine CMV also drives an intense virus-specific T cell response [41] and here studies reveal that 12 months of valaciclovir is required for significant suppression of virus-specific CD8 + T cell immunity [32] although MCMV-specific inflationary T cells have a half-life of only 45–60 days [42]. In contrast, the half-life of CMV and EBV-specific T cell populations within humans is considerably longer [26,43]. As such, the 6-month treatment period used within our study may be insufficient for substantial modulation of the virus-specific adaptive immune response. Although the magnitude of the virus-specific immune response was not altered by valaciclovir we were interested to see if any variation was noted in the phenotype of these cells, potentially reflecting loss of recent antigen stimulation as seen in HIV infection following anti-retroviral therapy [23,44], but again no impact was seen.

The CMV replication cycle proceeds in three stages: the immediate-early, early and late stages, whilst valaciclovir acts only at the late stage. As such, an important consideration for interpretation of our findings is that much of the virus-specific T cell response could be directed against peptides derived from genes expressed only during the immediate early or early phase of replication. Indeed, continuous surveillance by CD8 + T cells recognizing immediate-early antigenic epitopes during latent murine CMV infection blocks the transcriptional activity of viral genes downstream of that gene. Further, the ablation of a peptide from *IE1* can increase transcription of genes that are normally silenced [45] and was the basis for a model that unifies viral gene expression during latency with inflationary CD8 T cell responses [46]. A further notable observation is that immunodominant peptides from immediate early proteins can repress CD8 + T cell responses to peptides from early viral genes that are expressed even within the same latently infected cell [47,48]. Although our study focussed largely on epitopes encoded by IE1 or UL83 (pp65), recent evidence shows that even pp65 is expressed with immediate early kinetics [49] with immune recognition soon after infection [50]. Given these observations an intervention strategy that targets an immediate early gene, potentially with an antisense oligonucleotide against IE2 such as Fomivirsen, may have greater potential in suppressing the global CMV-specific T cell repertoire.

Our observations may also provide insight into the mechanisms that underlie maintenance of the CMV viral genome. The virus does not integrate into host DNA and, as no clear mechanism for tethering of episomal CMV DNA during latent expression has been established, it remains somewhat unclear how the viral genome is maintained across the lifecourse [51]. One suggestion is that intermittent episodes of productive viral replication are required to sustain lifetime persistence but our observation that 6 months of blockade of viral replication late in the lytic cycle has no discernible effects on virus load argues somewhat against this mechanism.

Two features of the CMV and EBV-specific cellular immune response were striking. Firstly, there was marked heterogeneity within individual donors and this variation in virus-host setpoint has been previously noted [6]. Whilst the genetic and environmental determinants of this profile are unclear, factors such as the initial viral load at primary infection [52] and host genetic polymorphism may be important [53]. The second feature was the profound stability of the virus-specific immune response over the 12-month observation period. Indeed, this cohort represents one of the most intensively studied prospective analyses of EBV or CMV-specific immune responses and shows that cross sectional assessment of immune response is representative of long-term profile. Assessment of general physical and mental health was also not impacted by therapy and may again reflect a minimal impact of herpesvirus infection on this measure within immunocompetent people.

Measurement of haematological and biochemical values over the study period did not reveal unexpected features. A notable finding was an increase in the mean cell volume of red cells during treatment, which correlated strongly with valaciclovir dose. This association has been reported previously and resolves rapidly following drug withdrawal [36]. The mechanism is unknown although it may potentially reflect a relative reduction in erythroblast proliferation rate, potentially related to the purine nucleoside analogue activity of the drug.

An epidemiological association between an inflated herpesvirus-specific immune response and impaired health outcome in older people has been seen in several studies [54,55]. Although this study failed so show any significant suppression of this immune response during medium-term valaciclovir therapy, a range of more potent anti-viral drugs have been developed and could themselves be tested for their utility in this setting.

## Materials and methods

### Ethics statement

Ethical approval was given by the East Midlands-Leicester Central Research Ethics Committee, study number 11/H0406/10. Formal written consent was obtained from all subjects prior to study entry.

### Study design

The study was single blind with subjects and clinicians aware of allocation but laboratory staff unaware of treatment assignment. Potentially eligible participants were identified using patient searches of GP clinical systems prior to postal invitation. Interested participants attended a baseline clinic where informed consent was taken prior to eligibility ascertainment.

A blood sample was taken to assess CMV serostatus, HLA genotype and the CMV-specific T cell immune response using intracellular cytokine (CD4^+^) and HLA-peptide tetramer (CD8^+^) analysis. Donors who were CMV seropositive with HLA-A*01:01, A*02:01, B*07:02 or B*08:01 genotype, and in whom the CD4^+^ and CD8^+^ CMV-specific immune response were both at least 0.2% of the total CD4^+^ and CD8^+^ T cell pools respectively, were eligible for enrolment and invited back for recruitment. At that point they were re-consented for treatment and 12 months of follow up. An estimated glomerular filtration rate (eGFR) >50ml/min was required for study entry.

Following recruitment, patients were randomised into four treatment groups with blood and urine samples taken prior to treatment; at monthly intervals whilst on treatment; and at 3 and 6 months following cessation of treatment. Donors were allocated randomly into four groups comprising either no treatment (n = 10) or valaciclovir at a dose of 500mg twice daily (n = 7), 1gm twice daily (n = 10) or 1gm four times daily (n = 11) for 6 months.

Patients completed a clinical questionnaire at study entry and then at each time point of study. EQ-5D-5L scores were determined at each timepoint (https://euroqol.org/information-and-support/euroqol-instruments/eq-5d-5l).

### Antibody titre against CMV and EBV

Peripheral blood mononuclear cells (PBMCs) were isolated together with serum and plasma.CMV serostatus was determined using ELISA [56] and antibody responses against EBV EBNA-1 and VCA. Briefly, Maxisorp plates were coated overnight with either recombinant EBNA-1, (1.0ug/ml, Abcam), or VCA-p18 (0.125ug/ml, RayBiotech), diluted in 0.1M, or 0.2M Carbonate Buffer (Sigma), respectively. Following washing (PBS + 0.05% Tween-20) and blocking (2% BSA in wash buffer), plasma diluted 1:5000 and 1:200 respectively, in blocking buffer, was added in duplicate. An 8-point standard curve was produced by serial dilution of a characterised standard added in duplicate on each plate. Bound IgG antibody was detected with anti-IgG-HRP and visualised with TMB. Plates were analysed at 450 and 660nm using a BioRad iMark microplate reader. Assays were tested, and baseline established, in respect of two commercial assay kits (Epstein Barr Virus EBNA-1 IgG ELISA kit, Abnova Corp and EB-VCA-IgG ELISA Kit, FineTest, Wuhan Fine Biotech co Ltd).

### T cell immune response against CMV and EBV

HLA genotype was defined by PCR and HLA-peptide tetramer staining and/or intracellular cytokine staining was utilized to quantify the CD8^+^ CMV-specific T cell response [57] (S2 Fig). CMV and EBV specific T cells were detected using HLA class I-peptide tetramers restricted through HLA-A1, A2, B7 and B8 (Table 2). CMV-specific CD4 + T cells were detected using HLA class II-peptide tetramers utilizing DR7, DR52, DQ6 (from NIH tetramer core facility) and DR15 (from ProImmune) and CMV immunodominant peptides [56] (Table 2).

**Table 2:** Peptides from CMV and EBV used in construction of HLA-peptide tetramers.

	CMV			EBV		
HLA-restriction	Peptide epitope	Antigen	Label	Peptide epitope		Label
HLA-A*01:01	YSEHPTFTSQY	pp65	YSE			
	VTEHDTLLY	pp50 (UL44)	VTE			
HLA-A*02:01	NLVPMVATV	pp65	NLV	GLCTLVAML	BMLF1	GLC
	VLEETSVML	IE-1	VLE	CLGGLLTMV	LMP-2A	CLG
				YVLDHLIVV	BRLF1	YVL
HLA-B*07:02	RPHERNGFTVL	pp65	RPH	RPPIFIRRL	EBNA3A	RPP
	TPRVTGGGAM	pp65	TPR	RPRATWIQEL	BaRF1	RPR
HLA-B*08:01	QIKVRVDMV	IE-1	QIK	RAKFKQLL	BZLF1	RAK
	ELRRKMMYM	IE-1	ELR	FLRGRAYGL	EBNA3A	FLR
	ELKRKMIYM	IE-1	ELK			
HLA-DRB1-*07:01 (DR7)	DYSNTHSTRYV	gB	DYS			
HLA-DRB1*15:01 (DR15)	MSIYVYALPLKMLNI	pp65	MSI			
HLA-DRB3*02:02 (DR52b)	AGILARNLVPMVATV	pp65	AGI			
HLA-DQB1*06:02 (DQ6)	LLQTGIHVRVSQPSL	pp65	LLQ			

HLA class I-restricted nonamer peptide tetramers for detection of CMV-specific CD8^+^ T cells were YSE and VTE presented by HLA-A*01:01; NLV and VLE presented by HLA*02:01; RPH and TPR presented by HLA*07:02; and QIK, ELR and ELK presented by HLA*08. Comparable reagents for detection of EBV-specific CD8^+^ T cells were GLC, CLG and YVL presented by HLA*A02:01; RPP and RPR presented by HLA*07:02; and RAK and FLR presented by HLA*08. HLA class II-restricted peptide tetramers for detection of CMV-specific CD4^+^ T cells were DYS presented by HLA*DR07:02; MSI presented by DR15; AGI presented by DR52b; and LLQ presented by DQ6 [57].

Intracellular cytokine staining was used to quantify the CD4^+^ CMV-specific T cell response following stimulation with viral lysate ([57]; S3 Fig). Antibody reagents used anti-Human CD4-PerCP-Cy5.5 (eBioScience 45–0049); anti-Human CD3-Amcyan (BD Biosciences 339186); anti-Human IFNγ-FITC (BD Biosciences 340449); anti-Human CD28-ECD (Beckman Coulter 6607111); anti-Human CD27-APCeFluor780 (eBioscience 47–0279) and Live/Dead Fixable Violet Stain (Invitrogen L34955).

Flow cytometry on whole blood samples was used to detect CD4^+^CD28^-^ and CD8^+^CD28^-^ T cells using anti-Human CD4-PercP-Cy5.5 (eBioscience 45–0049); Anti-Human CD3-AmCyan (BD Biosciences 339186); Anti-Human CD28-ECD (Beckman Coulter 6607111); Anti-Human CD45-FITC (eBioscience 11–9459); Anti-Human CD8-APC (eBioscience 17–0086); CytoCount Control Beads (Alere S236630) and FACS Lysing Solution (BD Biosciences 349202).

For determination of the number of total memory T cells, absolute counts of CD4+ and CD8 + T cells were established from whole blood samples (S2 Fig). The proportion of memory T cells was subsequently determined from phenotypic characterisation and applied to the absolute cell counts to determine the absolute count of memory T cells.

### Determining CMV/EBV genome load in PBMCs

Genomic DNA was isolated from 1x10^6^ PBMC pellets using a DNeasy Blood and Tissue Kits (Qiagen) according to the manufacturer’s manual book. Quantitative PCR assays were carried out to amplify EBV BALF5, HCMV-UL54 and cellular beta-2-microglobulin sequences to determine the EBV/HCMV genome load [58,59]. All standards and samples were tested in triplicate, and the data were analyzed using ABI Prism 7700 Sequence Detection System (PE Biosystems).

One reference plasmid containing the sequence that primers bind was used to generate standard curve for the Q-PCR. Standard curves were used to determine the copy numbers of EBV and CMV. The copy number of β2m was used to determine the cell input in each sample (assuming two copies per cell). Finally the viral load was calculated and shown as copy number per thousands PBMCs (CMV) and per million PBMCs (EBV).

### Primer and probe sequences

*EBV BALF5 gene* Forward primer: 5′ AGTCCTTCTTGGCTAGTCTGTTGAC 3′*;* Reverse primer: 5′ CTTTGGCGCGGATCCTC 3′; Probe: 5′ (FAM) CATCAAGAAGCTGCTGGCGGCCT 3′.
*Beta-2 microglobulin gene* Forward Primer: 5′ GGAATTGATTTGGGAGAGCATC 3′*;* Reverse Primer: 5′ CAGGTCCTGGCTCTACAATTTACTAA 3′; Probe: 5′ (VIC) AGTGTGACTGGGCAGATCATCCAGCTTC 3′.
*HCMV DNA polymerase* Forward primer: 5’-GCCGATCGTAAAGAGATGAAGAC 3’; reverse primer: 5’ CTCGTGCGTGTGCTACGAGA 3’; Probe: 5’ (FAM)-AGTGCAGCCCCGACCATCGTTC

## Supporting information

S1 Table Consort Diagram of Recruitment. (DOCX)

S2 Table Primary and secondary outcome results for the on-treatment period. (DOCX)

S3 Table Primary and secondary outcome results for the whole study period. (DOCX)

S1 Fig Enumeration of global CD4+ and CD8 + memory T cells during therapy. (DOCX)

S2 Fig Flow cytometry gating strategy to identify T cells subsets. (DOCX)

S3 Fig Flow cytometry gating strategy to identify CD4 + T cells responding to CMV lysate stimulation. (DOCX)
