# Supplementary figures and images for: Valaciclovir therapy for secondary suppression of immune response to herpesviruses: An exploratory study
Source: PLoS Pathog. 2025 Dec 29;21(12):e1013803. doi: 10.1371/journal.ppat.1013803 (PMC12768413; doi:10.1371/journal.ppat.1013803)

**
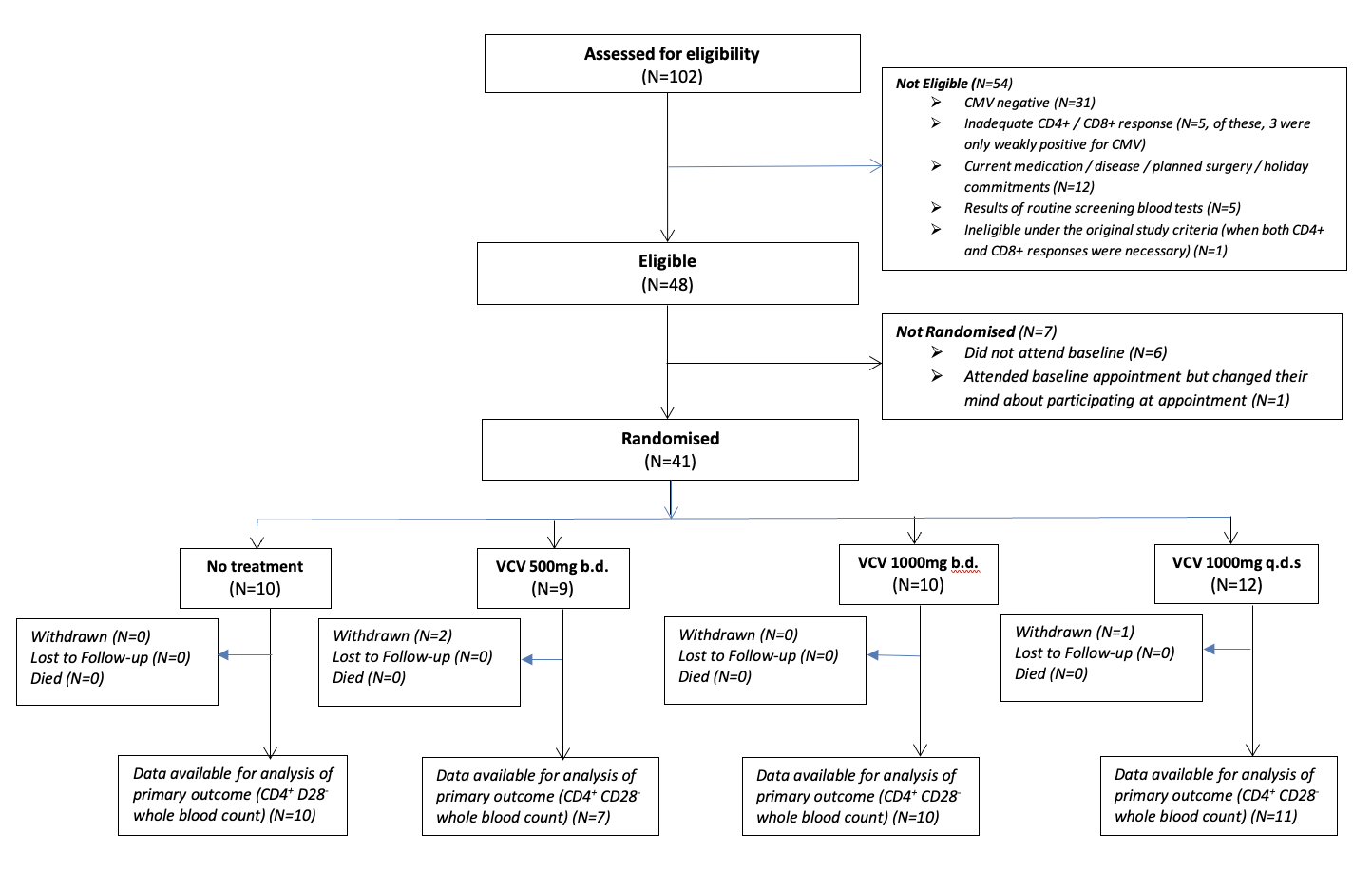
**

**Supplementary Table 1 Consort Diagram of Recruitment**

Supplement: S1 Table — (DOCX) [file ppat.1013803.s001.docx]

#
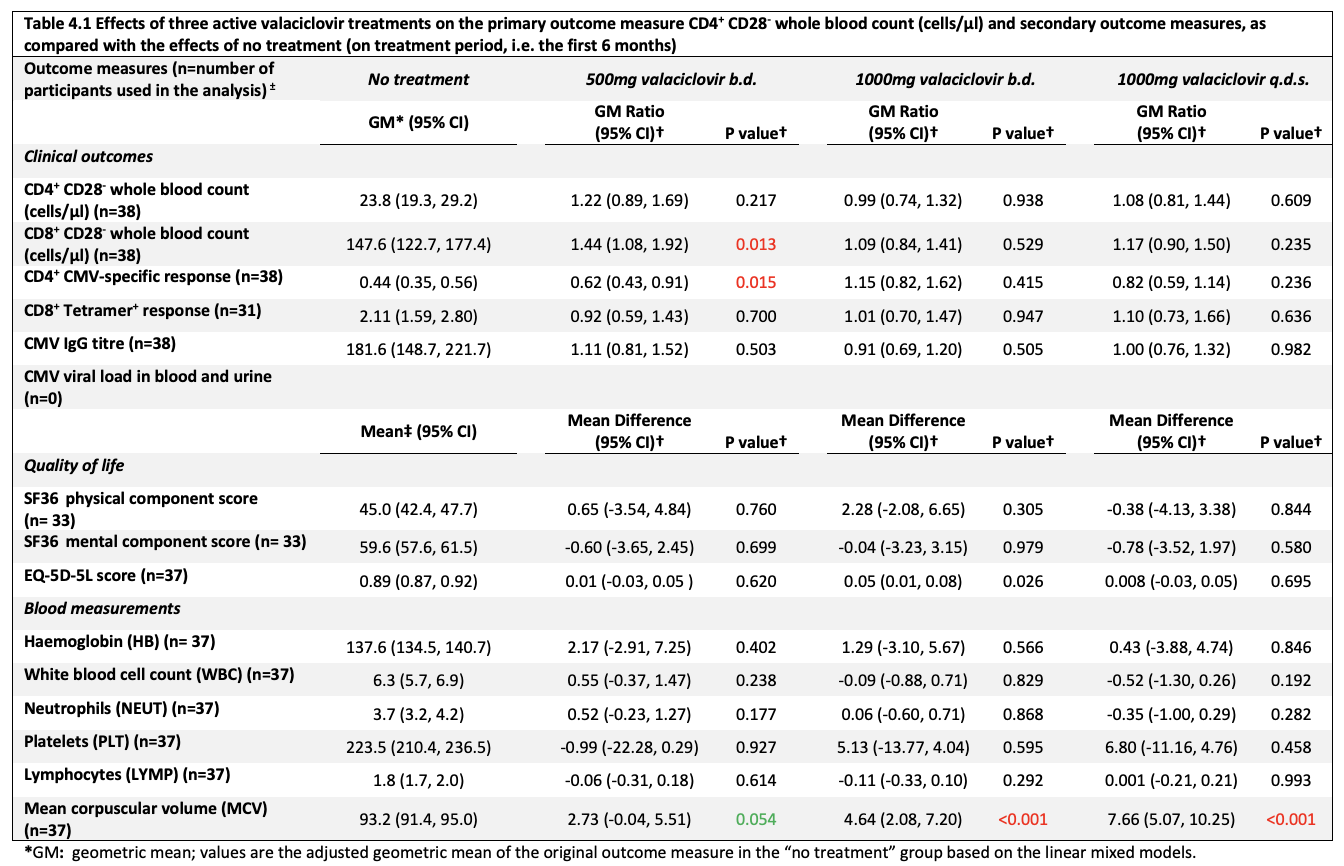


# Supplementary Table 2 Primary and secondary outcome results for the on-treatment period

Supplement: S2 Table — (DOCX) [file ppat.1013803.s002.docx]

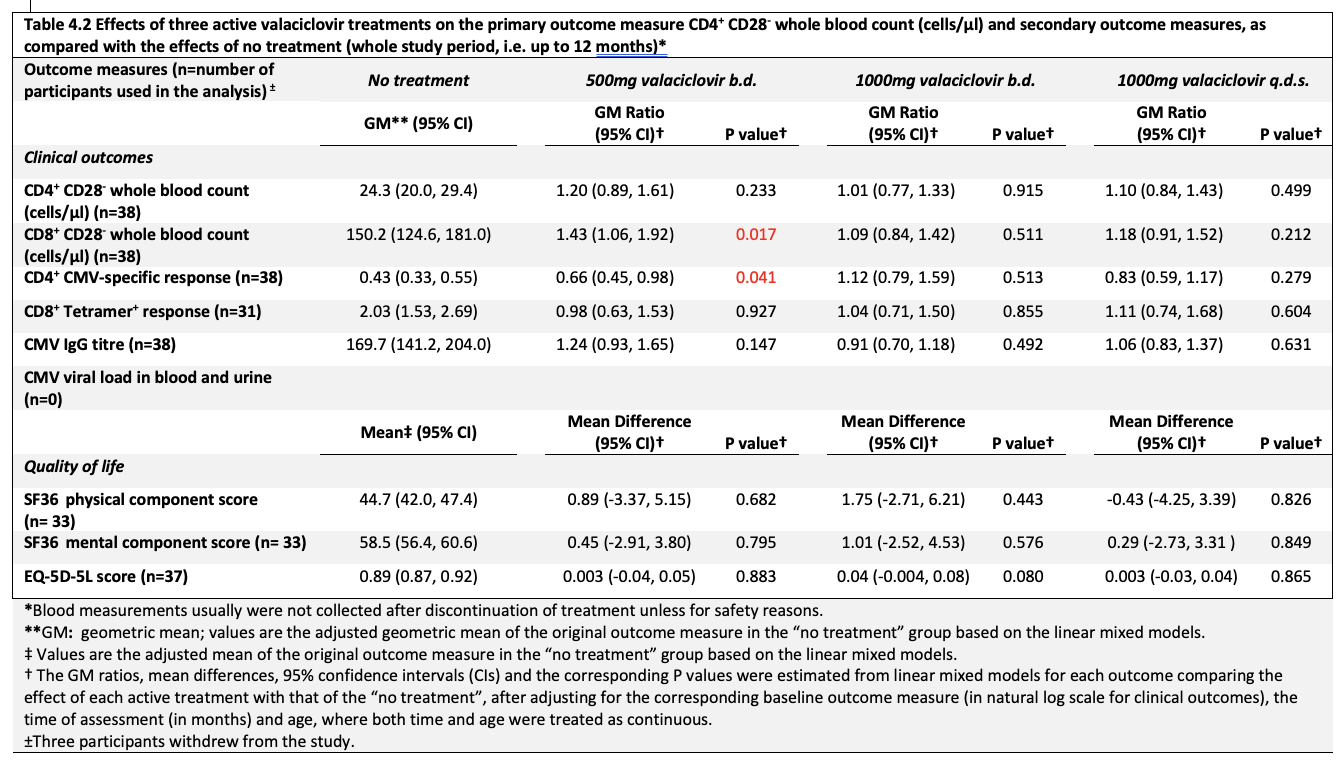


**Supplementary Table 3 Primary and secondary outcome results for the whole study perio****d**

Supplement: S3 Table — (DOCX) [file ppat.1013803.s003.docx]
