# Supplementary material for: Valaciclovir therapy for secondary suppression of immune response to herpesviruses: An exploratory study
Source: PLoS Pathog. 2025 Dec 29;21(12):e1013803. doi: 10.1371/journal.ppat.1013803 (PMC12768413; doi:10.1371/journal.ppat.1013803)
Supplement: S1 Fig — (DOCX) [file ppat.1013803.s004.docx]

**
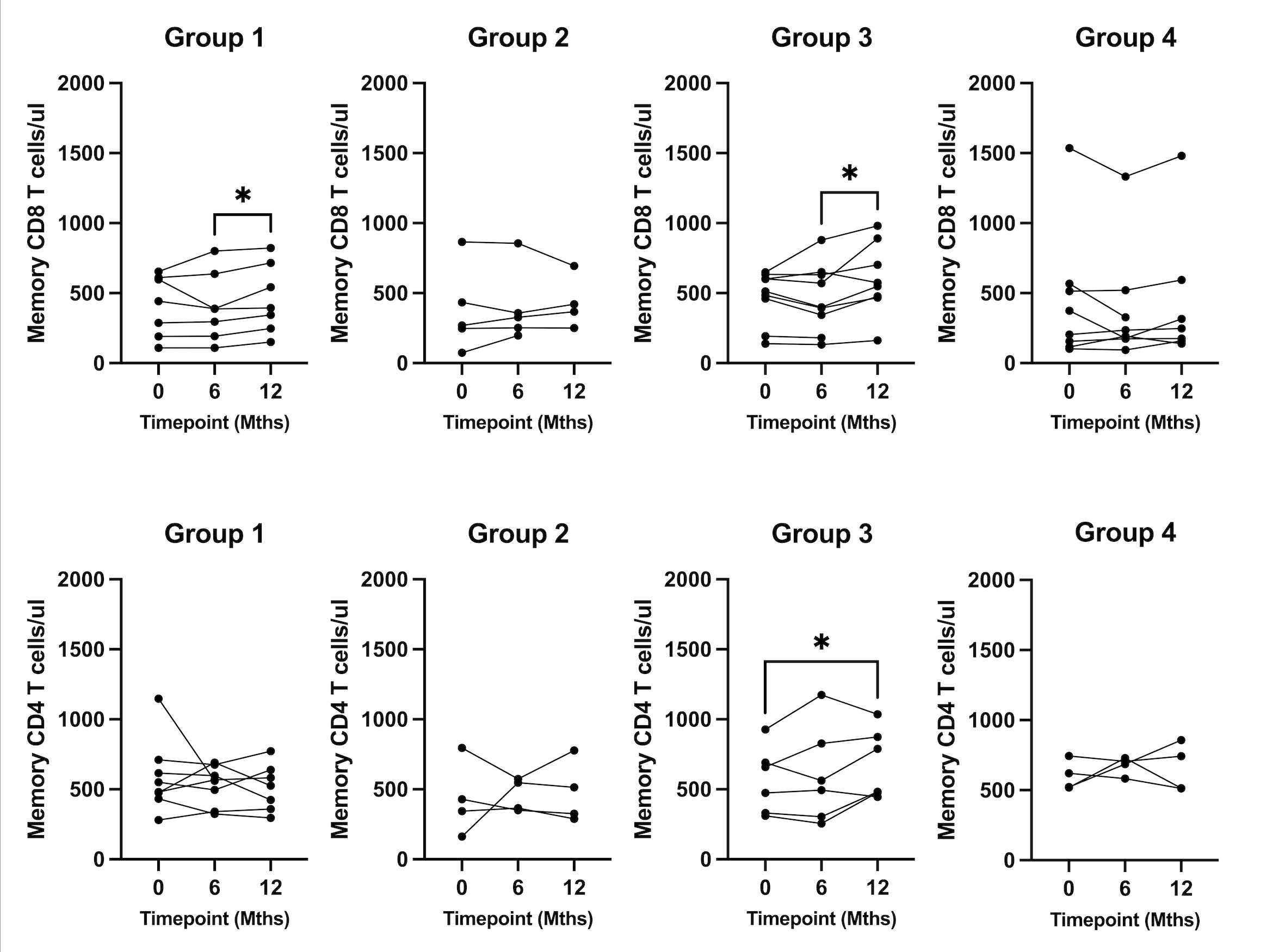
**

**Supplementary Figure 1: Enumeration of global memory T cell populations across the study period**.

Absolute counts of CD8+ (upper) and CD4+ (lower) memory T cells at commencement of study (0 mths), end of treatment (6 mths) and end of study follow up (12 mths) within the four study groups.
