# Supplementary material for: Valaciclovir therapy for secondary suppression of immune response to herpesviruses: An exploratory study
Source: PLoS Pathog. 2025 Dec 29;21(12):e1013803. doi: 10.1371/journal.ppat.1013803 (PMC12768413; doi:10.1371/journal.ppat.1013803)
Supplement: S2 Fig — (DOCX) [file ppat.1013803.s005.docx]

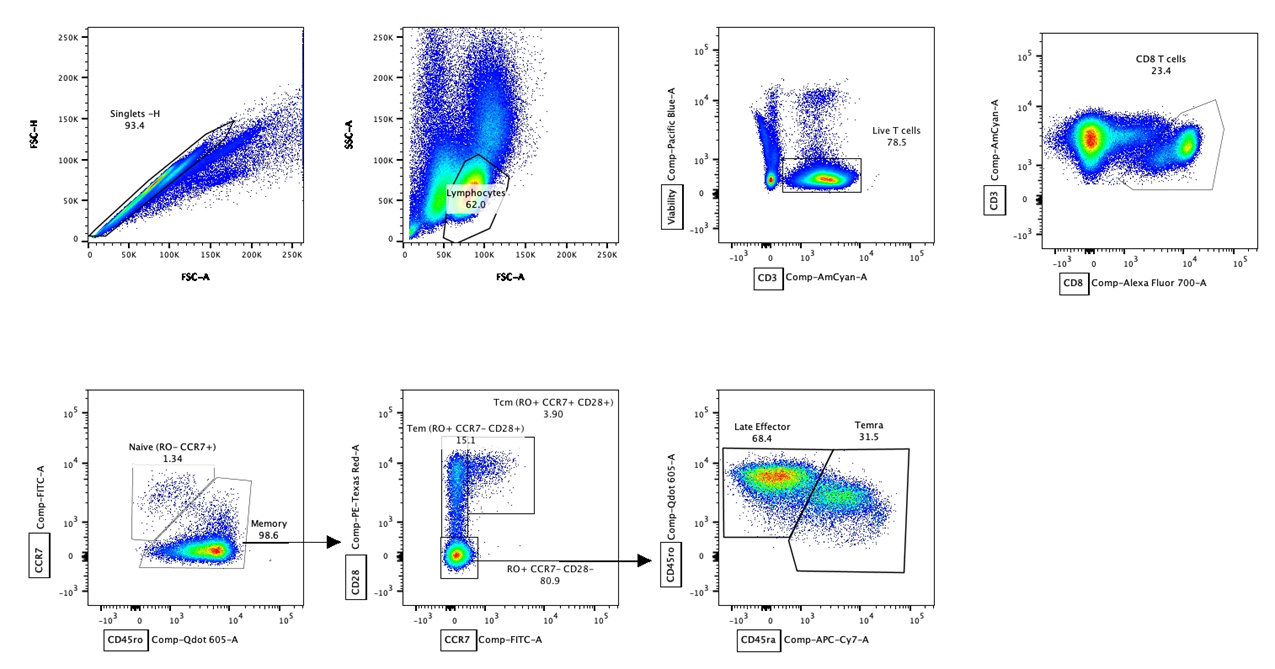


**Supplementary Figure 2: Gating strategy implemented to identify T cells subsets**

Analysis of flow cytometric data to identify: A) single cells, B), Lymphocytes C) Live cells, D) CD8+ T cells, (E) Naïve and memory CD8+ T cells and (F) Effector CD8+ T cells (G) Temra CD8+ T cells.
