# Supplementary material for: Valaciclovir therapy for secondary suppression of immune response to herpesviruses: An exploratory study
Source: PLoS Pathog. 2025 Dec 29;21(12):e1013803. doi: 10.1371/journal.ppat.1013803 (PMC12768413; doi:10.1371/journal.ppat.1013803)
Supplement: S3 Fig — (DOCX) [file ppat.1013803.s006.docx]

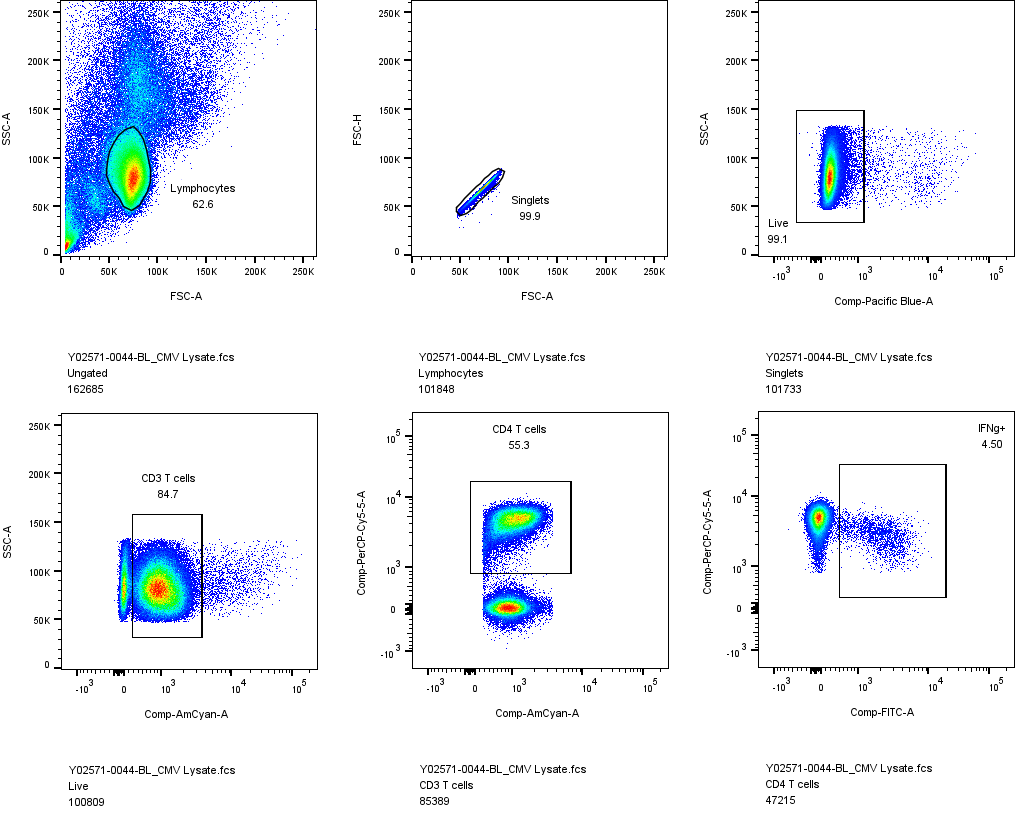


**Supplementary Figure 3: Gating strategy to identify CD4+ T cells responding to CMV lysate stimulation**

Analysis of flow cytometric data to identify: A) Lymphocytes, B) single cells, C) live cells, D) CD3+ T cells, (E) CD4+ T cells and (F) IFN-γ producing CD4+ T cells.
